# Throwing cold water on muscle growth: A systematic review with meta‐analysis of the effects of postexercise cold water immersion on resistance training‐induced hypertrophy

**DOI:** 10.1002/ejsc.12074

**Published:** 2024-02-05

**Authors:** Alec Piñero, Ryan Burke, Francesca Augustin, Adam E. Mohan, Kareen DeJesus, Max Sapuppo, Max Weisenthal, Max Coleman, Patroklos Androulakis‐Korakakis, Jozo Grgic, Paul A. Swinton, Brad J. Schoenfeld

**Affiliations:** ^1^ Department of Exercise Science and Recreation Applied Muscle Development Laboratory CUNY Lehman College Bronx New York USA; ^2^ Institute for Health and Sport Victoria University Melbourne Victoria Australia; ^3^ Department of Sport and Exercise School of Health Sciences Robert Gordon University Aberdeen UK

**Keywords:** cold application, cooling, cross‐sectional area, fat‐free mass, lean mass, recovery strategies

## Abstract

The purpose of this paper was to systematically review the literature and perform a meta‐analysis of the existing data on the effects of postexercise cold water immersion (CWI) coupled with resistance training (RT) on gains in measures of muscle growth. To locate relevant studies, we comprehensively searched the PubMed/MEDLINE, Scopus, and Web of Science databases. A total of 8 studies met the inclusion criteria; all investigated CWI as the means of cold application. Preliminary analyses conducted on noncontrolled effect sizes provided strong evidence of hypertrophic adaptations with RT that were likely to be at least small in magnitude (SMD_0.5_ = 0.36 [95% CrI: 0.10–0.61]; *p* (>0) = 0.995, *p* (>0.1) = 0.977). In contrast, noncontrolled effect sizes provided some evidence of hypertrophic adaptations with CWI + RT that were likely to be small to negligible in magnitude (SMD_0.5_ = 0.14 [95% CrI: −0.08–0.36]; *p* (>0) = 0.906, *p* (>0.1) = 0.68). The primary analysis conducted on comparative effect sizes provided some evidence of greater relative hypertrophic adaptations with RT compared to CWI + RT (cSMD_0.5_ = −0.22 [95% CrI: −0.47 to 0.04]) with differences likely to be greater than zero (*p* (<0) = 0.957) and of at least a small magnitude of effect (*p* (<−0.1) = 0.834). Meta‐regression did not indicate a potential moderation effect of training status ($\beta_{\text{Trained}:{\text{Untrained}}_{0.5}}$ = −0.10 [95% CrI: −0.65 to 0.43] *p* < 0) = 0.653). In conclusion, based on the current data, the application of CWI immediately following bouts of RT may attenuate hypertrophic changes. Given the overall relatively fair to poor quality of the studies examined, the results of the current study should be interpreted with some caution.

## Highlights

- Application of CWI immediately following bouts of RT may attenuate muscle hypertrophy in both trained and untrained individuals.
- The quality of evidence comparing RT versus CWI + RT is generally fair to poor, indicating a need for higher quality studies on the topic to draw stronger inferences.

## 1 INTRODUCTION

Practitioners employ a variety of strategies to attenuate the fatigue and discomfort of resistance training (RT) or physical competition, or in an attempt to improve performance‐defined measures of recovery. Of these strategies, extreme temperature exposure in various forms (e.g., sauna, cold and hot water immersion, cryotherapy, and phase‐change material) have been found to reduce the severity of muscle soreness (Crystal et al., 2013), perceived fatigue (Wang et al., 2021; Xiao et al., 2023), and time to recovery (Roberts et al., 2014). Notably, despite the target of cold therapy often‐cited as being a reduction of acute postexercise inflammation (McPhee & Lightfoot, 2017; Thorpe, 2021), there is some evidence that cold therapy may not actually reduce biological markers of inflammation to a greater extent than low‐intensity cycling (Peake et al., 2017) nor improve recovery from eccentric exercise‐induced muscle damage (Tseng et al., 2013). Of the various cold therapy strategies, cold water immersion (CWI), generally practiced by immersing the torso and limbs or individual limbs in water of <15^o^C for 10–20 min following an exercise bout (Broatch et al., 2018), has been found to improve recovery for certain types of subsequent athletic or training performance (Broatch et al., 2018; Versey et al., 2013; Xiao et al., 2023) though perhaps not all (Xiao et al., 2023). Based on recent meta‐analytic work, the application of CWI after a bout of high‐intensity exercise may confer a positive effect on various recovery‐related outcomes (Moore et al., 2022). It should be noted that performance outcomes in CWI research are influenced by the timing of assessment, nature of preceding exercise, and type of CWI protocol; thus, findings of these studies should be interpreted cautiously.

Although athletes commonly use CWI to enhance recovery and acutely improve exercise performance (Pointon & Duffield, 2012; Versey et al., 2013), the same might not hold true for chronic adaptations to RT. While there seem to be little or no negative effects of postexercise CWI on endurance training adaptations (Ihsan et al., 2021), CWI following RT may blunt gains in absolute strength and muscular power according to a series of recent narrative and meta‐analytical reviews (Broatch et al., 2018; Chaillou et al., 2022; Grgic, 2023; Malta et al., 2021; Petersen & Fyfe, 2021). However, no known meta‐analysis has been conducted on the effect of post‐exercise cold application on RT‐induced muscle hypertrophy. This gap in the literature should be considered given the evidence showing differential effects of CWI on strength versus hypertrophy. Specifically, a recent study reported that CWI attenuated gains in muscle size but not strength even though the data were collected in the same cohort (Fyfe et al., 2019).

There are various mechanistic reasons that suggest CWI may have detrimental effects on longitudinal skeletal muscle accretion. Most notably, CWI has been reported to acutely attenuate post‐RT mechanistic target of rapamycin complex 1 signaling (Fyfe et al., 2019), ribosome biogenesis (Figueiredo et al., 2016), muscle protein synthesis (MPS) (Fuchs et al., 2020), satellite cell activity (Roberts et al., 2015), and increases in circulating testosterone and cytokines (Earp et al., 2019)—responses which may, to varying degrees, negatively impact muscular adaptations (Crewther et al., 2006; Figueiredo & McCarthy, 2019; Koh & Pizza, 2009; Kraemer & Ratamess, 2005; Ogasawara & Suginohara, 2018). The purpose of this paper was to systematically review the literature and perform a meta‐analysis of the existing data on the effects of postexercise cooling coupled with RT on gains in measures of muscle hypertrophy.

## 2 METHODS

We conducted this review in accordance with the guidelines of the “Preferred Reporting Items for Systematic Reviews and Meta‐Analyses” (PRISMA) (Page et al., 2021). The study was preregistered on the Open Science Framework (https://osf.io/gx69b); supplemental files can be downloaded at: https://osf.io/cdm9w/.

### 2.1 Search strategy

To locate relevant studies, we comprehensively searched the PubMed/MEDLINE, Scopus, and Web of Science databases using the following Boolean search syntax: (“cold water immersion” OR “CWI” OR “cryotherapy” OR “cryo” OR “cryostimulation” OR “cryochamber” OR “ice bath*” OR “ice‐bath*” OR “ice water bath” OR “ice‐water bath” OR “cold exposure” OR “cold application” OR “cold plunge” OR “cold stress” OR “cold treatment” OR “post‐exercise cooling” OR “post exercise cooling” OR “cooling therapy*” OR “contrast‐water therapy” OR “contrast water therapy”) AND (“resistance training” OR “resistance exercise” OR “weight lifting” OR “weightlifting” OR “strength exercise” OR “strength training” OR “strengthening” OR “resistive exercise” OR “resistive training”) AND (“muscle hypertrophy” OR “muscular hypertrophy” OR “muscle growth” OR “muscular growth” OR “muscle mass” OR “muscle development” OR “muscular development” OR “muscle volume” OR “lean body mass” OR “fat‐free mass” OR “fat free mass” OR “lean mass” OR “muscle fiber” OR “muscle size” OR “muscular size” OR “myofiber” OR “myofibre” OR “muscle fiber” OR “muscle thickness” OR “cross‐sectional area” OR “cross sectional area”). In addition, we screened the reference lists of articles retrieved and applicable review papers to uncover any additional studies that might meet inclusion criteria as per Greenhalgh and Peacock (Greenhalgh & Peacock, 2005).

The search process was carried out separately by 3 researchers (AP, MW, and KD). The initial search consisted of screening all titles and abstracts for studies potentially meeting inclusion/exclusion criteria. For papers deemed potentially relevant, full texts were evaluated and decisions were then made as to whether a given study warranted inclusion. Any disputes the search team could not resolve were settled by a fourth researcher (BJS). The search was finalized on February 24^th^, 2023.

### 2.2 Inclusion criteria

We included studies that satisfied the following criteria: (a) had a randomized design (either within‐ or between‐group design) and directly compared CWI + RT versus RT with a sham or active/passive recovery (both with and without adjuvant dietary interventions) for estimates of changes in proxies of lean/muscle mass using a validated measure (dual‐energy X‐ray absorptiometry [DXA], bioelectrical impedance analysis, magnetic resonance imaging [MRI], computerized tomography [CT], ultrasound [US], muscle biopsy, or limb circumference measurement) in healthy adults; (b) involved at least 2 RT sessions per week for a duration of at least 4 weeks (NOTE: In our preregistration, we indicated a minimum duration of 6 weeks, but after perusing the data, we decided to accept studies with a duration of at least 4 weeks given evidence of appreciable hypertrophy at this timepoint (DeFreitas et al., 2011) and then subanalyze data by study length); (c) published in a peer‐reviewed English language journal or on a pre‐print server. We excluded studies that utilized participants with comorbidities that might impair muscle hypertrophy responses (musculoskeletal disease/injury/cardiovascular impairments).

### 2.3 Data extraction

For each included study, 2 researchers (RB and AM) independently extracted and coded the following data: Author name(s), title and year of publication, sample size, participant characteristics (i.e., sex, training status, and age), description of the training intervention (i.e., duration, volume, load, frequency, proximity to failure, and body region), method for hypertrophy assessment (i.e., DXA, MRI, CT, US, biopsy, and circumference measurement), and mean pre‐ and post‐study measures of proxies of lean/muscle mass values with corresponding standard deviations. In cases where measures of changes in lean/muscle mass were not reported, we attempted to contact the corresponding author(s) to obtain the data. If unattainable, we extracted the data from graphs (when available) via online software (https://automeris.io/WebPlotDigitizer/). To account for the possibility of coder drift, a third researcher (FA) recoded 30% of the studies, which were randomly selected for assessment (Cooper et al., 2009). Per case agreement was determined by dividing the number of variables coded the same by the total number of variables. Acceptance required a mean agreement of 0.90. Any discrepancies in the extracted data were resolved through discussion and mutual consensus of the coders.

### 2.4 Methodological quality

As noted in the preregistration, we originally planned to use the Downs and Black assessment tool (Downs & Black, 1998) to assess study quality. However, after discussion with the research team, it was determined that the proposed tool was too generic to properly evaluate the complexities of longitudinal RT research. Thus, we developed an alternative tool specifically designed to assess the quality (both in terms of risk of bias as well as transparency of reporting) of longitudinal RT interventions. We named the tool as follows: Standards Method for Assessment of Resistance Training in Longitudinal Designs (SMART‐LD).

The SMART‐LD tool consists of 20 questions that address the following aspects of a study's methodology: general (items 1–2), participants (items 3–7), training program (items 8–11), outcomes (items 12–16), and statistical analyses (17–20). Each item in the checklist is given 1 point if the criterion is satisfied or 0 points if the criterion is not satisfied. The values of all questions are summed with the final total used to classify studies as follows: “good quality” (16–20 points); “fair quality” (12–15 points); or “poor quality” (≤11 points).

As per Resnick et al. (2000), we established content validity of the tool by initially creating a list of items that addressed the primary aspects of repeated measures RT protocols (participants, program, outcomes, and statistics). Our team of five experienced researchers reached a consensus on the content and wording of the items included in the tool. We then sought input from colleagues who provided additional feedback on areas of relevance and ambiguity. After addressing the input from our colleagues, we sent the tool to four independent researchers, all experienced with carrying out longitudinal RT trials, to rate the relevance of the items on a scale of 1 (not very relevant) to 4 (very relevant). The mean relative rating for all items between the 4 raters was 3.56 (89%) and no item was rated “not very relevant,” indicating the tool has high relevance for evaluating the quality of longitudinal RT designs. An overview of the items included in the SMART‐LD tool and explanation of the grading criteria for each item can be found at: https://osf.io/nhva2/.

We thus employed the SMART‐LD tool as the primary quality assessment of studies included in this meta‐analysis. Four reviewers (AP, MC, RB, and PAK) independently rated each study; any disputes were resolved by majority consensus. Given our initial intention to employ the Downs and Black checklist as noted in the preregistration, we also carried out a quality assessment of studies using this tool. Three reviewers (AP, MS, and FA) independently rated each study; any disputes were resolved by majority consensus.

### 2.5 Statistical analyses

A Bayesian framework was chosen over a frequentist approach as it provides a more flexible modeling that enables results to be presented intuitively by reporting subjective probabilities (Kruschke & Liddell, 2018). As muscle growth was measured using different methods in the included studies, the primary analysis was conducted using comparative standardized mean difference effect sizes (cSMD) calculated from direct comparisons between CWI + RT and RT. To provide additional context, preliminary analyses were conducted with noncontrolled standardized mean difference effect sizes (SMD) to determine whether the CWI + RT and RT programs tended to result in hypertrophic adaptations. Three‐level random‐effects Bayesian hierarchical models were used to pool effect sizes and model the mean comparative effect, variance within studies, variance between studies, and covariance of multiple outcomes reported in the same study (i.e., multiple outcomes and/or single outcome reported at multiple time points following baseline). Within‐study variances were calculated using standard distributional assumptions (Morris 2008; Morris & DeShon, 2002) with adjustment for cross‐over designs where required (Madeyski & Kitchenham, 2018). Within‐study variances are dependent on pre‐post correlations (Morris 2008) that are generally not reported. Rather than specify a single correlation value, this was estimated but constrained using an informative prior distribution. Similarly, informative prior distributions were used for the comparative effect sizes based on previous meta‐analysis data (Swinton & Murphy, 2022). Sensitivity analyses were conducted using weakly informative prior distributions (see supplemental file S1). A sensitivity analysis was also conducted by removing studies that applied localized cooling to a small amount of muscle mass due to the potential differences this strategy could create compared to other more standard practices.

Inconsistency in models was described by comparing variances across the three levels. Inferences from all analyses were performed on posterior samples generated using the Hamiltonian Markov Chain Monte Carlo method and through credible intervals (CrIs) and calculated probabilities (*p*). Interpretations were based on medians (e.g., cSMD_0.5_), range of values within CrIs, and calculation of probabilities that the magnitude of the pooled mean effect size exceeded qualitative thresholds (i.e., small, medium, and large) specific to strength and conditioning interventions (Swinton et al., 2022; Swinton & Murphy, 2022). For noncontrolled effect sizes the small, medium, and large thresholds selected were +0.10, +0.35, and +0.70 (Swinton et al., 2022) with values of ±0.10, ±0.30, and ±0.50 used for comparative effect sizes (34). Meta‐regression or subgroup analyses were performed where sufficient data were available, including a minimum of 4 data points per category level or 10 data points for continuous variables Fu et al., 2011). Small‐study effects (publication bias, etc.) were visually inspected with funnel plots and quantified with a multilevel extension of Egger's regression‐intercept test (Fernandez‐Castilla et al., 2021). Analyses were performed using the *R* wrapper package *brms* interfaced with *Stan* to perform the sampling (Bürkner, 2017). Full model details, including prior distributions for all meta‐analyses, are presented in the supplementary file tables (S1) with summary descriptions presented in text.

## 3 RESULTS

### 3.1 Descriptive data

A total of eight interventions met the inclusion criteria (see supplemental file S2); all employed CWI as the means of cold application (see Table 1). The duration of the included studies ranged from 4 to 12 weeks. All studies included young adults (aged 20–26 years) of which 7 studies included only male participants (Fyfe et al., 2019; Horgan et al., 2023; Ohnishi et al., 2004; Roberts et al., 2015; Wilson et al., 2021; Yamane et al., 2006; Yamane et al., 2015) and 1 study included both male and female participants (Poppendieck et al., 2021). Four studies examined resistance‐trained participants (Horgan et al., 2023; Poppendieck et al., 2021; Roberts et al., 2015; Wilson et al., 2021) and the others employed untrained participants (Fyfe et al., 2019; Ohnishi et al., 2004; Yamane et al., 2006; Yamane et al., 2015). Six studies incorporated a parallel group design (Fyfe et al., 2019; Ohnishi et al., 2004; Roberts et al., 2015; Wilson et al., 2021; Yamane et al., 2006; Yamane et al., 2015), and the other two employed a within‐subject crossover design (Horgan et al., 2023; Poppendieck et al., 2021). All the RT sessions were performed 2–3 times per week. Two studies solely focused on training handgrip (40,41), 1 study solely focused on training the wrist flexors (Yamane et al., 2015), 3 studies trained just the lower body (Poppendieck et al., 2021; Roberts et al., 2015; Wilson et al., 2021), and the other 2 employed full body training protocols (Fyfe et al., 2019; Horgan et al., 2023). Only 2 studies reported intensity of effort with 1 reporting that participants trained to failure (Poppendieck et al., 2021) and 1 reporting participants trained with various percentages of repetition maximum (Horgan et al., 2023) and none of the other studies reporting proximity to failure. Three studies reported that training was directly supervised (Horgan et al., 2023; Poppendieck et al., 2021; Roberts et al., 2015), while the other 5 did not report whether training was supervised or unsupervised (Fyfe et al., 2019; Ohnishi et al., 2004; Wilson et al., 2021; Yamane et al., 2006; Yamane et al., 2015). Three studies exposed only upper limbs to CWI (Ohnishi et al., 2004; Yamane et al., 2006; Yamane et al., 2015), 2 studies exposed only lower limbs to CWI (Roberts et al., 2015; Wilson et al., 2021), and 3 studies exposed participants to full‐body CWI (Fyfe et al., 2019; Horgan et al., 2023; Poppendieck et al., 2021). Three studies applied CWI for 10 min (Poppendieck et al., 2021; Roberts et al., 2015; Wilson et al., 2021), 2 studies applied CWI for 15 min (Fyfe et al., 2019; Horgan et al., 2023), and 3 applied CWI for 20 min (Ohnishi et al., 2004; Yamane et al., 2006; Yamane et al., 2015). Water temperature was 10^o^C for 6 of the studies (Fyfe et al., 2019; Ohnishi et al., 2004; Roberts et al., 2015; Wilson et al., 2021; Yamane et al., 2006; Yamane et al., 2015), 15^o^C for 1 of the studies (Horgan et al., 2023), and between 14 and 15^o^C for the last (Poppendieck et al., 2021). CWI was administered 3 min post‐RT in 2 studies (Yamane et al., 2006; Yamane et al., 2015), 5 min post‐RT in 2 studies (Fyfe et al., 2019; Roberts et al., 2015), and 15 min post‐RT in 2 studies (Horgan et al., 2023; Wilson et al., 2021), while 2 studies only specified that CWI followed the last set of exercise (Ohnishi et al., 2004) and immediately followed each training session (Poppendieck et al., 2021). Three studies reported total lean body mass via DXA (Fyfe et al., 2019; Horgan et al., 2023; Wilson et al., 2021) with only 1 reporting distinct upper body and lower body measurements (Fyfe et al., 2019). For site‐specific measures of hypertrophy, 2 studies used biopsy of the vastus lateralis to analyze type I and type II muscle fibers (Fyfe et al., 2019; Roberts et al., 2015), 1 study used US of the vastus medialis (Poppendieck et al., 2021), 1 study measured leg circumference (Poppendieck et al., 2021), 1 study used MRI of the quadriceps (Roberts et al., 2015), 1 study used US of the forearm (Yamane et al., 2006), 1 study used US for wrist flexors (Yamane et al., 2015), and 2 studies measured forearm circumference (Ohnishi et al., 2004; Yamane et al., 2015).

**TABLE 1:** Summary of the methods of included studies.

Study	Sample	Design	RT protocol	CWI protocol	Hypertrophy measure	Duration
Fyfe et al. (2019)	8 untrained men	Random assignment to 1 of 2 groups: (1) RT + CWI; (2) RT + PASS	TB protocol performed 3 days/wk consisting of 3–5 sets per exercise at either 20RM or 12RM	TB CWI for 15 min at 10^o^C beginning 5 min post‐RT	‐ DXA: UB, LB, TLM ‐ Biopsy: VL	7 weeks
Horgan et al. (2023)	17 trained men	Random order assignment to 1 of 3 conditions with a crossover design: (1) RT + CWI; (2) RT + SS	TB supervised protocol performed 2 days/wk consisting of 2–4 sets per exercise at 66%–87% 1RM or body weight with 1 set completed every 4 min; core exercises consisted of 3 sets with 15s interset rest intervals	TB CWI for 15 min at 15^o^C beginning 15 min post‐RT. SS group performed static stretching at 23°C	‐ DXA: TLM	4 weeks
Ohnishi et al. (2004)	16 untrained men	Random assignment to 1 of 2 groups: (1) RT + CWI; (2) RT + PASS	Unilateral handgrip protocol performed 3 days/wk consisting of 3 sets at 8RM	UL CWI for 20 min at 10^o^C following RT	‐ CIR: Forearm	6 weeks
Poppendieck et al. (2021)	11 trained men and women (*M* = 9; *W* = 2)	Random order assignment to 1 of 2 conditions with a crossover design: (1) RT + CWI; (2) RT + PASS	LB supervised protocol performed 2 days/wk consisting of 3 sets per exercise at 10RM with 3 min interset rest intervals	TB CWI for 10 min at 14–15^o^C immediately following RT	‐ US: VM ‐ CIR: Leg	8 weeks
Roberts et al. (2015)	21 trained men	Random assignment to 1 of 2 groups: (1) RT + CWI; (2) RT + ACT	LB supervised protocol performed 2 days/wk consisting of 3–6 sets per exercise at 8–12RM for machine exercises, 20%+ PTBM for WWL, body weight for CDJ, and 50% of lunge load for SESJ, SLJ, and CBJ with 1 min interset rest intervals and 3 min interexercise rest intervals	LL CWI for 10 min at 10^o^C beginning 5 min post‐RT. ACT group performed 10 min low intensity stationary cycling	‐ Biopsy: VL ‐ MRI: QUAD	12 weeks
Wilson et al. (2021)	13 trained men	Random assignment to 1 of 2 groups: (1) RT + CWI; (2) RT + SI	LB protocol performed 2 days/wk consisting of 2 blocks.	LL CWI for 10 min at 10^o^C beginning 15 min post‐RT. SI group were told they received extra leucine supplementation	‐ DXA: TLM	8 weeks
			‐ Strength block consisted of 3–4 sets per exercise at 4–6RM except for JS (2 reps @load eliciting peak power) ‐ Power block consisted of 5 sets at: final strength block weight (1/4 squat for 2 reps and JS for 3 reps), body weight (box jumps for 3 reps), same weight as JS (squat jumps for 3 reps), and 30‐cm box (drop jumps for 3 reps)			
Yamane et al. (2006)	16 untrained men	Random assignment to 1 of 2 groups: (1) RT + CWI; (2) RT + PASS	Bilateral handgrip protocol performed 3 days/wk consisting of 3 sets of handgrip ergometer exercise at 8RM (70%–80% 1RM) with 2 min inter‐set rest intervals	UL CWI for 20 min at 10^o^C beginning 3 min post‐RT	US: Forearm	4 weeks
Yamane et al. (2015)	14 untrained men	Random assignment to 1 of 2 groups: (1) RT + CWI; (2) RT + PASS	Unilateral WF protocol performed 3 days/wk consisting of 5 sets of 8 reps of wrist flexion ergometer exercise 70%–80% 1RM with 2 min interset rest intervals	UL CWI for 20 min at 10^o^C beginning 3 min post‐RT	‐ US: WF ‐ CIR: Forearm	6 weeks

Abbreviations: ACT, active recovery; CBJ, countermovement box jumps; CDJ, countermovement drop jumps; CIR, circumference; DXA, dual‐energy x‐ray absorptiometry; JS, jump shrugs; LB, lower body; LL, lower limbs; MRI, magnetic resonance imaging; PASS, passive recovery; PTBM, pretraining body mass; QUAD, quadriceps; SESJ, slow eccentric squat jumps; SI, sham intervention; SLJ, split lunge lumps; SS, static stretching; TB, total body; TLM, total lean mass; UB, upper body; UL, upper limb; US, ultrasound; VL, vastus lateralis; VM, vastus medialis; WF, wrist flexor; WWL, weighted walking lunges.

### 3.2 Meta‐analyses

**FIGURE 1:**
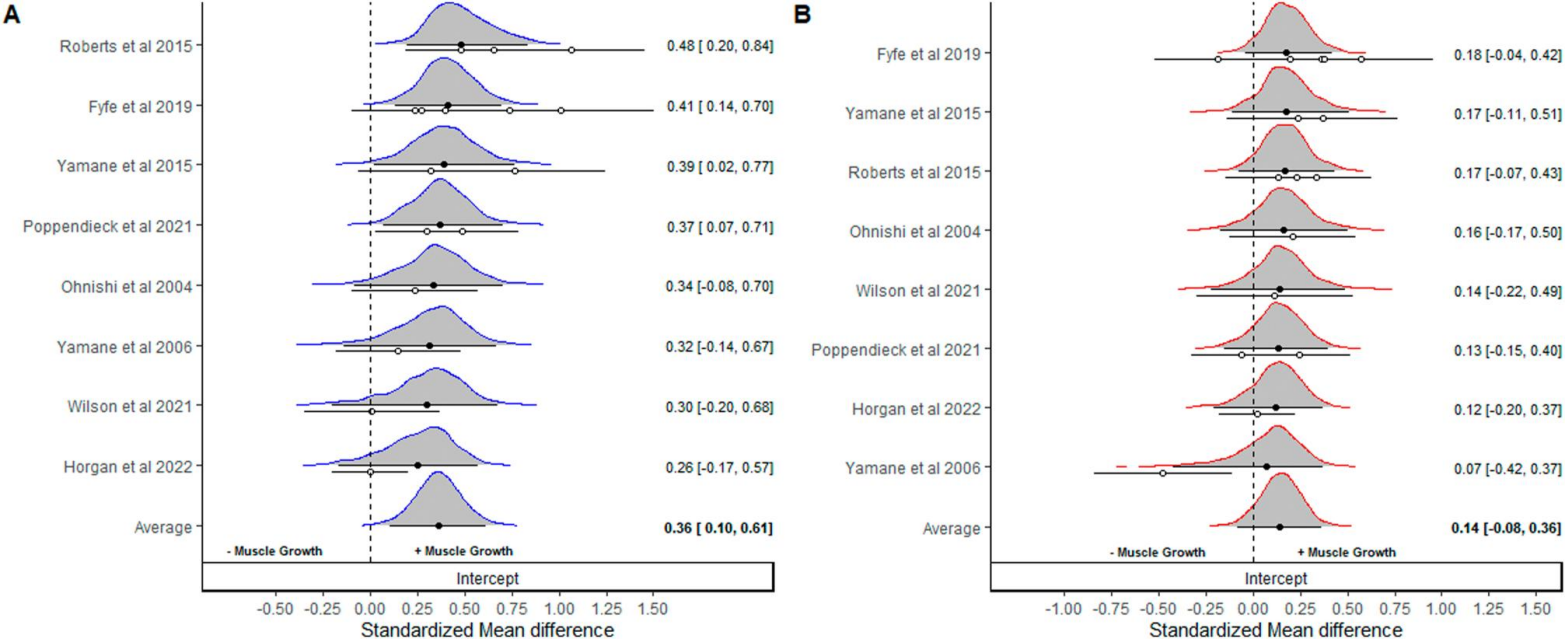
Bayesian forest plots illustrating pooling of noncontrolled standardized mean difference effect sizes for resistance training (A) and with cold water immersion with resistance training (B). Positive values indicate muscle growth and negative values indicate a reduction in muscle following intervention. Distributions represent “shrunken estimates” based on all effects sizes included, the random effects model fitted and borrowed information across studies to reduce uncertainty. Black circles and connected intervals represent the median value and 95% credible intervals for the shrunken estimates. White circles and intervals represent the raw estimates and sampling variance calculated directly from study data. Bottom distributions illustrate uncertainty in the pooled means.

Preliminary analyses conducted on noncontrolled effect sizes provided strong evidence of hypertrophic adaptations with RT that were likely to be at least small in magnitude (SMD_0.5_ = 0.36 [95% CrI: 0.10–0.61]; *p* (>0) = 0.995, *p* (>0.1) = 0.977); Figure 1). In contrast, noncontrolled effect sizes provided some evidence of hypertrophic adaptations with CWI + RT that were likely to between small and negligible in magnitude (SMD_0.5_ = 0.14 [95% CrI: −0.08–0.36]; *p* (>0) = 0.906, *p* (>0.1) = 0.68); Figure 1). Full model details, including information of prior distributions, are presented in supplementary file S1 (Table S1).

**FIGURE 2:**
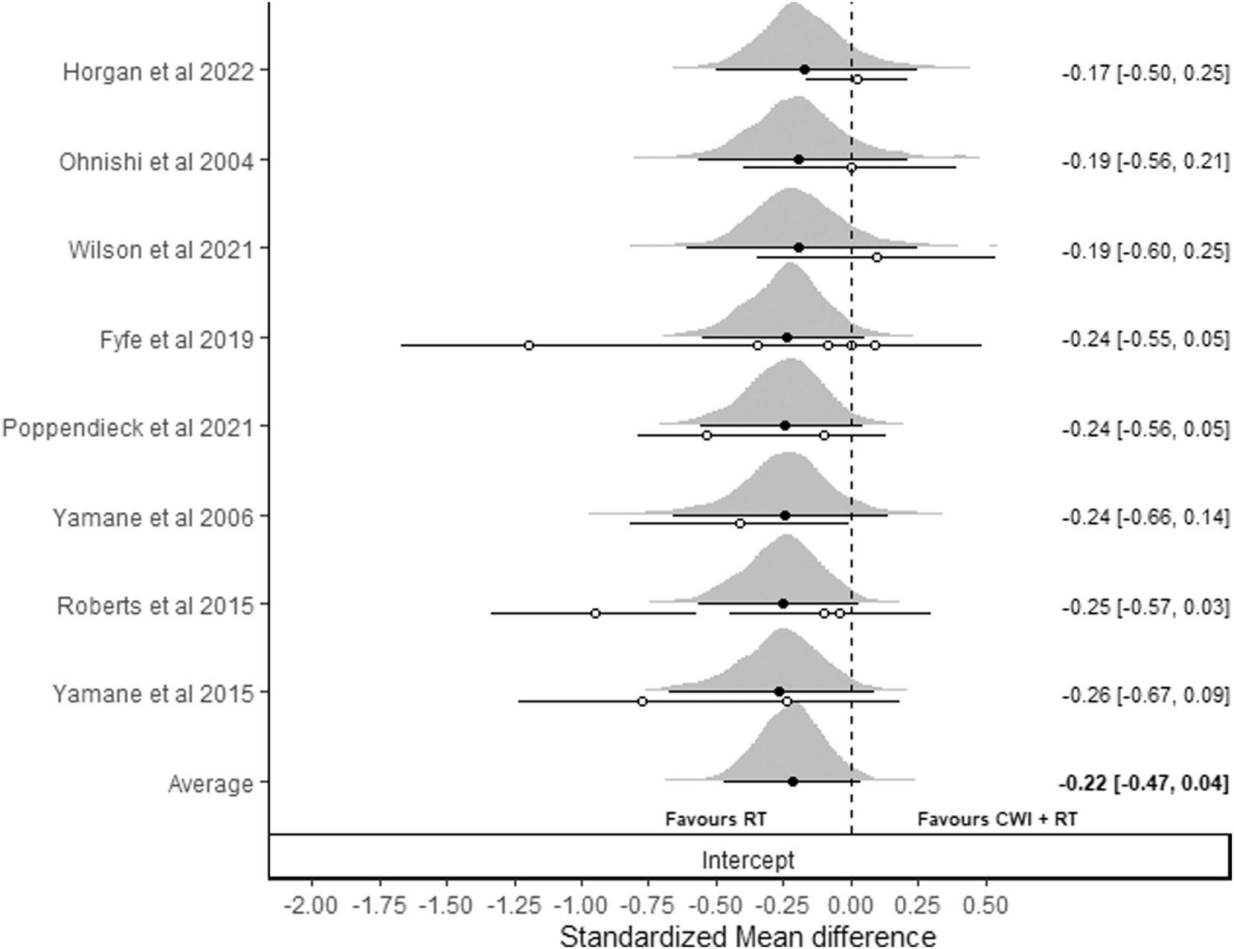
Bayesian forest plot illustrating pooling of comparative standardized mean difference effect sizes directly comparing resistance training (RT) and cold water immersion with resistance training (CWI + RT). Positive values favor cold water immersion and resistance training (CWI + RT) and negative values favor resistance training (RT). Distributions represent “shrunken estimates” based on all effects sizes included, the random effects model fitted and borrowed information across studies to reduce uncertainty. Black circles and connected intervals represent the median value and 95% credible intervals for the shrunken estimates. White circles and intervals represent the raw estimates and sampling variance calculated directly from study data. Bottom distribution illustrates uncertainty in the pooled mean.

The primary analysis conducted on comparative effect sizes (Figure 2) provided some evidence of greater relative hypertrophic adaptations with RT compared to CWI + RT (cSMD_0.5_ = −0.22 [95% CrI: −0.47 to 0.04]) with differences likely to be greater than zero (*p* (<0) = 0.957) and of at least a small effect (*p* (<−0.1) = 0.834). Full model details, including information of prior distributions, are presented in supplementary file S1 (Table S2). A sensitivity analysis removing studies that applied localized cooling to a small amount of muscle mass (Ohnishi et al., 2004; Yamane et al., 2006; Yamane et al., 2015) had a trivial influence on findings with increased uncertainty concomitant with reduction in the amount of data available (CWI + RT (cSMD_0.5_ = −0.19 [95% CrI: −0.51 to 0.25]); differences were likely to be greater than zero (*p* (<0) = 0.882) and of at least a small effect (*p* [<−0.1] = 0.7115]).

Three meta‐regressions were conducted to investigate the potential moderation effect of intervention duration (Shorter: <8 weeks; Longer: ≥8 weeks), training status (Trained, Untrained), and training frequency (Two days per week, Three days per week). Limited evidence of a moderation effect was obtained for all factors with the CrIs demonstrating high uncertainty (Shorter: Longer 0.5 = −0.04 [95% CrI: −0.61 to 0.55], *p* (<0) = 0.570; Trained: Untrained 0.5 = −0.10 [95% CrI: −0.65 to 0.43], *p* (<0) = 0.653, Twodays: Threedays −0.15 [95% CrI: −0.76 to 0.41], *p* (<0) = 0.720). Full model details, including information of prior distributions, are presented in supplementary file S1 (Table S3).

**FIGURE 3:**
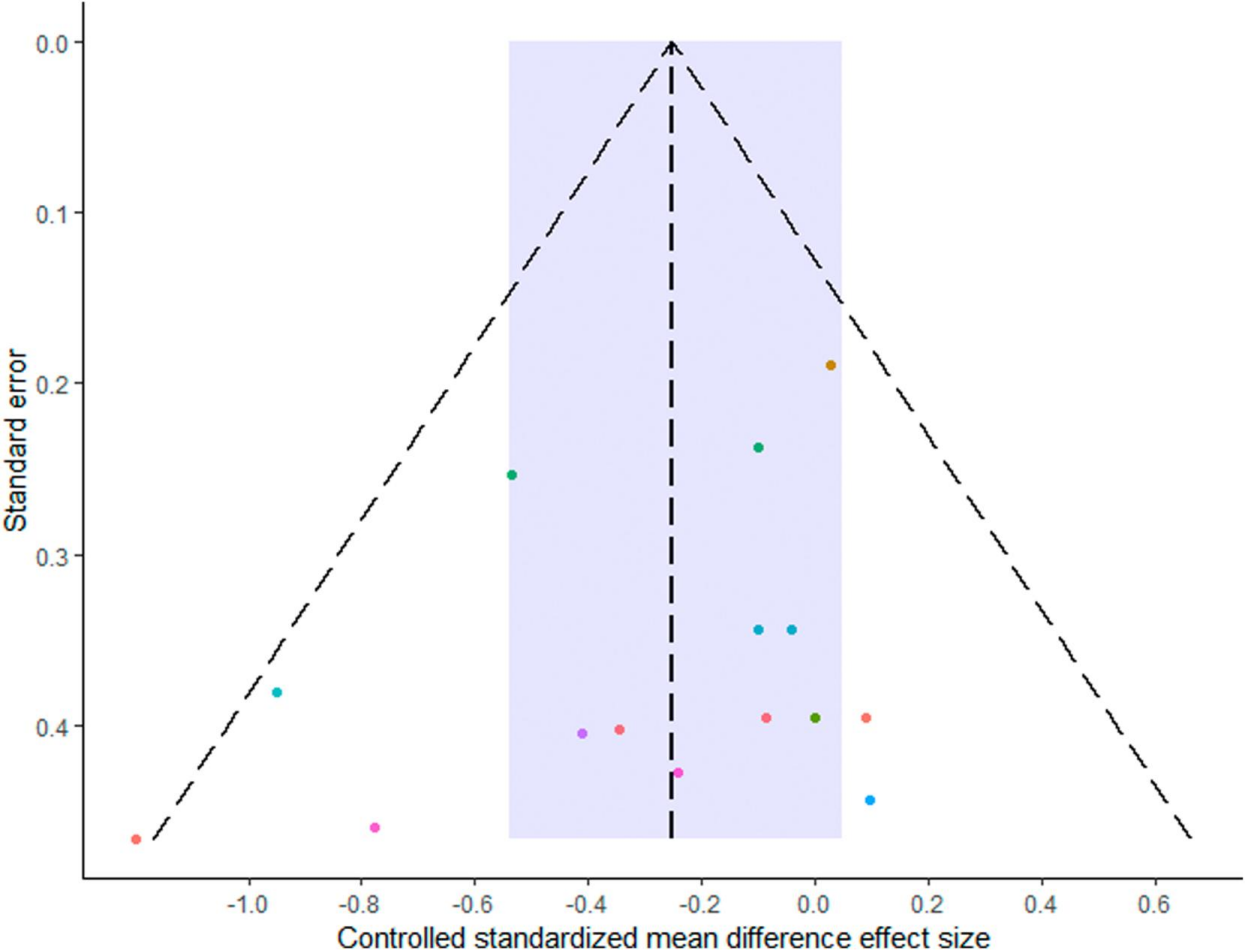
Funnel plot of all comparative effect sizes. Data are colored according to the individual studies. Blue region illustrates the pooled mean estimate and 95% credible interval.

Egger's regression intercept test produced wide intervals, and a visual inspection of the funnel plot (Figure 3) did not identify any small‐study‐related issues.

### 3.3 Study quality

A qualitative assessment of the studies via the SMART‐LD tool indicated a mean score of 9.8 out of a possible 20 points (range: 4–13 points). No studies were deemed to be of good quality, 3 studies were deemed to be of fair quality (Horgan et al., 2023; Fyfe et al., 2019; Roberts et al., 2015), and 5 studies were deemed to be of poor quality (Ohnishi et al., 2004; Poppendieck et al., 2021; Wilson et al., 2021; Yamane et al., 2006; Yamane et al., 2015).

A qualitative assessment of the studies via the Downs and Black checklist indicated a mean score of 18.4 out of a possible 29 points (range: 12–24 points). Two studies were deemed to be of good quality (Horgan et al., 2023; Roberts et al., 2015), 6 studies were classified as being of fair quality (Fyfe et al., 2019; Ohnishi et al., 2004; Poppendieck et al., 2021; Wilson et al., 2021; Yamane et al., 2006; Yamane et al., 2015), and no studies were found to be of poor quality.

## 4 DISCUSSION

This is the first known systematic review with meta‐analysis to examine the effects of CWI on RT‐induced skeletal muscle hypertrophy. Although research indicates that CWI does not completely prevent muscular gains, our results provide some evidence that it likely attenuates adaptations compared with RT alone. Based on our data, the application of CWI may result in at least a small magnitude of reduction in hypertrophy with the upper credible interval identifying a relatively low probability of a moderate detrimental effect. Subanalysis using meta‐regression provided a lack of evidence that training status altered the likely attenuation of muscle hypertrophy with CWI.

Our findings are consistent with data from acute studies, which have reported that CWI blunts the anabolic response to RT. For example, Fuchs et al. (2020) reported that CWI administered 20‐min after performance of lower body RT reduced MPS rates for up to 5 h postapplication. The researchers also demonstrated an impaired MPS response to CWI during prolonged RT (Fuchs et al., 2020). In addition, evidence indicates that the post‐RT exposure to CWI attenuates activation of transcriptional factors involved in ribosome biogenesis (Figueiredo et al., 2016) and suppresses satellite cell activity (Roberts, et al., 2015), both of which are purported to be important mediators of skeletal muscle hypertrophy (Brook et al., 2019). These alterations persisted for up to 48 h after application suggesting a prolonged deleterious effect.

While the scope of this analysis did not include mechanistic drivers of skeletal muscle accretion, potential physiological mechanisms behind attenuated hypertrophy outcomes following CWI have been proposed in previous research. One such hypothesis is that CWI alters the acute inflammatory response to RT, which has been implicated in the kinase domain of titan hypertrophy (Koh & Pizza, 2009). A reduction in inflammatory responses to RT could also conceivably attenuate reactive oxygen species production (Uchiyama et al., 2006) and associated activation of the mitogen‐activated protein kinase pathway (Roux & Blenis, 2004), thereby downregulating MPS (Takarada et al., 2000) and potential anabolism. To this point, some human trials have reported blunted inflammatory‐related cytokine responses to RT in the minutes and hours following cold exposure including inflammation markers, such as interleukin‐6 (IL‐6), tumor necrosis factor‐α, and plasma chemokine ligand 2 (Crystal et al., 2013; Earp et al., 2019). On the other hand, Peake et al. (2017) found similar acute blunting of proinflammatory cytokines between CWI and active recovery groups after a bout of intense RT. Similarly, Ahokas et al. (2020) reported little difference in inflammation markers following CWI and thermoneutral water immersion following high‐intensity sprinting and jumping. Overall, meta‐analytic data on the effects of CWI on inflammatory markers indicate that only C‐reactive protein is appreciably elevated following postexercise application (Hohenauer et al., 2015). The conflicting data call into question whether postexercise reductions in acute inflammatory responses induced by CWI play a role in altering muscle development.

It also is possible that CWI negatively affects anabolism via reductions in postexercise blood flow to the musculature, a potential impact that should not be overlooked as blood flow and nutrient‐dependent skeletal muscle proteolysis and MPS regulatory effects of insulin are well established (Fujita et al., 2006; Timmerman et al., 2010). In brief, studies have found that CWI may reduce blood flow to musculature exposed to RT compared to both thermoneutral water (Gregson et al., 2011) and whole‐body cryotherapy (Mawhinney et al., 2017) interventions. While the post‐RT anabolic window for muscle growth associated with MPS may not be as narrow as once thought (Schoenfeld & Aragon, 2018), an acute reduction in post‐RT nutrient delivery, and therefore an extended period of muscle protein catabolism and diminished maximal MPS capacity via CWI‐induced blood flow impairment is theoretically plausible. Whether these acute outcomes directly or indirectly affect long‐term hypertrophic adaptations remains undetermined.

Regarding the quality of the included studies, we employed 2 separate assessments to determine their risk of bias and transparency of reporting. The frequently used Downs and Black checklist indicated that the included studies were generally of good to fair quality. Alternatively, the SMART‐LD tool, created to specifically assess the quality of longitudinal RT research, indicated studies were generally of fair to poor quality. Based on these results, higher quality studies are needed to draw stronger inferences as to the effects of CWI on muscle development. Moreover, the discrepancies in results between the 2 tools indicate that commonly employed quality assessment methods may not be sufficient for evaluating longitudinal RT interventions.

### 4.1 Limitations

There are several limitations that must be acknowledged when attempting to draw evidence‐based conclusions from our analysis. First, the majority of studies included in this analysis lasted between 4 and 8 weeks with only one intervention exceeding 8 weeks (Roberts et al., 2015). Although there is evidence that the combination of CWI and RT modestly impairs measures of muscular hypertrophy, it is not clear if the comparison between groups would have varied over longer time frames.

Second, the heterogeneity of measurement methods between studies can be considered a limitation to the validity of the findings as the ability to draw inferences regarding the efficacy of a RT protocol is largely predicated on the assessment tools used (Haun et al., 2019). The studies included in this meta‐analysis employed a wide array of measurements, including biopsy, DXA, circumference, US, and MRI. However, direct imaging modalities (i.e., MRI, CT and US) have been shown to be more accurate for assessing hypertrophic adaptations compared to indirect modalities (i.e., DXA) (Delmonico et al., 2008; Levine et al., 2000). Moreover, biopsy shows high coefficients of variation (∼13%) for assessing fiber CSA (Horwath et al., 2021) indicating questionable reliability for this modality. Thus, future studies investigating the effects of CWI on muscular adaptations should seek to employ direct imaging methods either alone or in combination with other modalities. On the other hand, the fact that a variety of modalities indicate CWI impairs hypertrophy, even those less sensitive to detecting subtle changes in muscle mass, would seem to strengthen the confidence in our conclusions.

Third, the RT protocols varied greatly between studies, including total weekly training volume, frequency, and proximity to failure. Several of these studies included only exercises targeting smaller muscle groups during single‐joint movements, which may not accurately reflect the training programs of most athletes. Only 3 studies (Ohnishi et al., 2004; Yamane et al., 2006; Yamane et al., 2015) included exercises involving multijoint movements commonly seen in athletic settings. Furthermore, only 1 study (Fyfe et al., 2019) used whole‐body RT, which may be essential for determining if CWI has localized or systemic effects on RT adaptations.

Fourth, most of the included studies did not attempt to compile nutritional intake across the respective study periods. It is well‐documented that both total daily energy and protein consumption influences the hypertrophic response to RT (Aragon & Schoenfeld, 2020; Morton et al., 2017). Although the randomized designs would seemingly help to account for nutritional discrepancies between groups, the relatively small sample sizes of studies could have unduly influenced the responses in the respective groups. Thus, future studies should seek to account for dietary intake to ensure this variable does not confound results.

Fifth, all the included studies administered CWI therapy following every RT session using a similar approach (i.e., 10–20 min, <15 min following training, and 10–15^o^C), and there may be alternative CWI approaches to consider. Realistically, CWI therapy may be applied only intermittently throughout a certain period of time (e.g., a week or month) and does not necessarily have to be applied immediately following each training session. Therefore, it is possible that alternative approaches to CWI application might yield different results. Future research should look to establish consistent and ecologically valid standards as to the timing and frequency of CWI application to enhance the generalizability of findings.

Sixth, our results are specific to the use of CWI as a recovery strategy. We, therefore, cannot necessarily extrapolate findings to other cold application strategies, such as cryotherapy, which warrant further investigation as to their chronic effects on muscular adaptations.

Seventh, all protocols employed relatively moderate loads (>8RM) and modest training volumes. Horgan et al. (2023) speculated that CWI may help athletes maintain an “adaptive sweet‐spot” under high training loads. It is conceivable that during periods of intensive training with high loads and/or volumes that the beneficial effects of CWI for blunting chronic inflammation may outweigh other detrimental effects on hypertrophic mechanisms associated with the strategy. There was insufficient data to subanalyze this hypothesis; therefore, further research is needed with heavy loads and/or high volumes to determine whether such a phenomenon indeed exists.

Finally, the pooled subject population consisted primarily of young men; only 1 of the 8 studies (Poppendieck et al., 2021) involved female participants and no studies involved adolescents or older adults. Thus, our findings cannot necessarily be generalized to other populations. Given the influences that recovery, muscular sensitivity, and endocrine factors can have on RT adaptations (Baláš et al., 2020; Castellani & Young, 2016; Petrofsky & Laymon, 2009), future research should investigate the impact of cooling strategies on muscular hypertrophy across populations.

## 5 CONCLUSION

The current data provides evidence that the application of CWI immediately following bouts of RT may modestly attenuate gains in muscle hypertrophy. When considering the practical implications of these findings, it is important to note that the results of this analysis apply solely to CWI application within 15 min of exercise cessation, which may not accurately reflect ecologically valid scenarios where CWI is employed several hours post‐RT and/or implemented periodically rather than exclusively on RT days. It is unknown as to whether, or the degree to which, intermittent use of CWI or more time between RT sessions and CWI application may influence gains in muscle mass. Thus, individuals seeking to maximize muscle hypertrophy should avoid using CWI immediately following bouts of RT and further consider the frequency and timing of application. In addition, the current results suggest that RT in combination with CWI may still induce gains in muscle mass but to a lesser degree compared to RT alone. These findings may have practical implications for athletes looking to limit RT‐induced gains in muscle mass (e.g., distance runners). Further research is needed to understand the effects of different frequencies and timing strategies of CWI on RT‐induced muscular adaptations, especially in resistance trained individuals and endurance athletes.

## CONFLICT OF INTEREST STATEMENT

There is no conflict to disclose.

## Supporting information

Supplementary Material S1

Figure S1

## References

[ejsc12074-bib-0001] Ahokas, E. K. , H. Kyröläinen , A. A. Mero , S. Walker , H. G. Hanstock , and J. K. Ihalainen . 2020. “Water Immersion Methods Do Not Alter Muscle Damage and Inflammation Biomarkers after High‐Intensity Sprinting and Jumping Exercise.” European Journal of Applied Physiology 120(12): 2625–2634. 10.1007/s00421-020-04481-8. 32880050 PMC7674333

[ejsc12074-bib-0002] Aragon, Alan A. , and Brad J. Schoenfeld . 2020. “Magnitude and Composition of the Energy Surplus for Maximizing Muscle Hypertrophy: Implications for Bodybuilding and Physique Athletes.” Strength and Conditioning Journal 42(5): 79–86. 10.1519/ssc.0000000000000539.

[ejsc12074-bib-0003] Baláš, Jiří , Jan Kodejška , Dominika Krupková , and David Giles . 2020. “Males Benefit More from Cold Water Immersion during Repeated Handgrip Contractions Than Females Despite Similar Oxygen Kinetics.” The Journal of Physiological Sciences 70(1): 13–020. 10.1186/s12576-020-00742-5. 32138641 PMC7058574

[ejsc12074-bib-0004] Brms, Bürkner P. 2017. “An R Package for Bayesian Multilevel Models Using Stan.” Journal of Statistical Software 80(1): 1–28.

[ejsc12074-bib-0005] Broatch, James R. , Aaron Petersen , and David J. Bishop . 2018. “The Influence of Post‐exercise Cold‐Water Immersion on Adaptive Responses to Exercise: A Review of the Literature.” Sports Medicine 48(6): 1369–1387. 10.1007/s40279-018-0910-8. 29627884

[ejsc12074-bib-0006] Brook, Matthew Stewart , Daniel James Wilkinson , Ken Smith , and Philip James Atherton . 2019. “It's Not Just about Protein Turnover: The Role of Ribosomal Biogenesis and Satellite Cells in the Regulation of Skeletal Muscle Hypertrophy.” European Journal of Sport Science 19(7): 952–963. 10.1080/15367290.2019.1569726. 30741116

[ejsc12074-bib-0007] Castellani, John W. , and Andrew J. Young . 2016. “Human Physiological Responses to Cold Exposure: Acute Responses and Acclimatization to Prolonged Exposure.” Autonomic Neuroscience 196: 63–74. 10.1016/j.autneu.2016.02.009. 26924539

[ejsc12074-bib-0008] Chaillou, Thomas , Viktorija Treigyte , Sarah Mosely , Marius Brazaitis , Tomas Venckunas , and Arthur J. Cheng . 2022. “Functional Impact of Post‐exercise Cooling and Heating on Recovery and Training Adaptations: Application to Resistance, Endurance, and Sprint Exercise.” Sports Medicine Open 8(1): 37. 10.1186/s40798-022-00428-9. 35254558 PMC8901468

[ejsc12074-bib-0009] Cooper, H. , L. Hedges , and J. Valentine . 2009. The Handbook of Research Synthesis and Meta‐Analysis. 2nd ed. New York: Russell Sage Foundation.

[ejsc12074-bib-0010] Crewther, Blair , Justin Keogh , John Cronin , and Christian Cook . 2006. “Possible Stimuli for Strength and Power Adaptation: Acute Hormonal Responses.” Sports Medicine 36(3): 215–238. 10.2165/00007256-200636030-00004. 16526834

[ejsc12074-bib-0011] Crystal, Naomi J. , David H. Townson , Summer B. Cook , and Dain P. LaRoche . 2013. “Effect of Cryotherapy on Muscle Recovery and Inflammation Following a Bout of Damaging Exercise.” European Journal of Applied Physiology 113(10): 2577–2586. 10.1007/s00421-013-2693-9. 23873339

[ejsc12074-bib-0012] DeFreitas, Jason M. , Travis W. Beck , Matt S. Stock , Michael A. Dillon , and Paul R. Kasishke . 2011. “An Examination of the Time Course of Training‐Induced Skeletal Muscle Hypertrophy.” European Journal of Applied Physiology 111(11): 2785–2790. 10.1007/s00421-011-1905-4. 21409401

[ejsc12074-bib-0013] Delmonico, M. J. , M. C. Kostek , J. Johns , B. F. Hurley , and J. M. Conway . 2008. “Can Dual Energy X‐Ray Absorptiometry Provide a Valid Assessment of Changes in Thigh Muscle Mass with Strength Training in Older Adults?” European Journal of Clinical Nutrition 62(12): 1372–1378. 10.1038/sj.ejcn.1602880. 17684523

[ejsc12074-bib-0014] Downs, S. H. , and N. Black . 1998. “The Feasibility of Creating a Checklist for the Assessment of the Methodological Quality Both of Randomised and Non‐randomised Studies of Health Care Interventions.” Journal of Epidemiology & Community Health 52(6): 377–384. 10.1136/jech.52.6.377. 9764259 PMC1756728

[ejsc12074-bib-0015] Earp, Jacob E. , Disa L. Hatfield , Andrew Sherman , Elaine C. Lee , and William J. Kraemer . 2019. “Cold‐water Immersion Blunts and Delays Increases in Circulating Testosterone and Cytokines Post‐resistance Exercise.” European Journal of Applied Physiology 119(8): 1901–1907. 10.1007/s00421-019-04178-7. 31222379

[ejsc12074-bib-0016] Fernández‐Castilla, Belén , Lies Declercq , Laleh Jamshidi , S. Natasha Beretvas , Patrick Onghena , and Wim Van den Noortgate . 2021. “Detecting Selection Bias in Meta‐Analyses with Multiple Outcomes: A Simulation Study.” The Journal of Experimental Education 89(1): 125–144. 10.1080/00220973.2019.1582470.

[ejsc12074-bib-0017] Figueiredo, Vandré Casagrande , and John J. McCarthy . 2019. “Regulation of Ribosome Biogenesis in Skeletal Muscle Hypertrophy.” Physiology 34(1): 30–42. 10.1152/physiol.00034.2018. 30540235 PMC6383632

[ejsc12074-bib-0018] Figueiredo, Vandré C. , Llion A. Roberts , James F. Markworth , Matthew P. G. Barnett , Jeff S. Coombes , Truls Raastad , Jonathan M. Peake , and David Cameron‐Smith . 2016. “Impact of Resistance Exercise on Ribosome Biogenesis is Acutely Regulated by Post‐Exercise Recovery Strategies.” Physics Reports 4(2): e12670. 10.14814/phy2.12670. PMC4760384 26818586

[ejsc12074-bib-0019] Fu, Rongwei , Gerald Gartlehner , Mark Grant , Tatyana Shamliyan , Art Sedrakyan , Timothy J. Wilt , Lauren Griffith , et al. 2011. “Conducting Quantitative Synthesis when Comparing Medical Interventions: AHRQ and the Effective Health Care Program.” Journal of Clinical Epidemiology 64(11): 1187–1197. 10.1016/j.jclinepi.2010.08.010. 21477993

[ejsc12074-bib-0020] Fuchs, Cas J. , Imre W. K. Kouw , Tyler A. Churchward‐Venne , Joey S. J. Smeets , Joan M. Senden , Wouter D. van Marken Lichtenbelt , Lex B. Verdijk , and Luc J. C. van Loon . 2020. “Postexercise Cooling Impairs Muscle Protein Synthesis Rates in Recreational Athletes.” The Journal of Physiology 598(4): 755–772. 10.1113/jp278996. 31788800 PMC7028023

[ejsc12074-bib-0021] Fujita, Satoshi , Blake B. Rasmussen , Jerson G. Cadenas , James J. Grady , and Elena Volpi . 2006. “Effect of Insulin on Human Skeletal Muscle Protein Synthesis Is Modulated by Insulin‐Induced Changes in Muscle Blood Flow and Amino Acid Availability.” American Journal of Physiology. Endocrinology and Metabolism 291(4): E745–E754. 10.1152/ajpendo.00271.2005. 16705054 PMC2804964

[ejsc12074-bib-0022] Fyfe, Jackson J. , James R. Broatch , Adam J. Trewin , Erik D. Hanson , Christos K. Argus , Andrew P. Garnham , Shona L. Halson , Remco C. Polman , David J. Bishop , and Aaron C. Petersen . 2019. “Cold Water Immersion Attenuates Anabolic Signaling and Skeletal Muscle Fiber Hypertrophy, but Not Strength Gain, Following Whole‐Body Resistance Training.” Journal of Applied Physiology 127(5): 1403–1418. 10.1152/japplphysiol.00127.2019. 31513450

[ejsc12074-bib-0023] Greenhalgh, Trisha , and Richard Peacock . 2005. “Effectiveness and Efficiency of Search Methods in Systematic Reviews of Complex Evidence: Audit of Primary Sources.” BMJ 331(7524): 1064–1065. 10.1136/bmj.38636.593461.68. 16230312 PMC1283190

[ejsc12074-bib-0024] Gregson, Warren , Mark A. Black , Helen Jones , Jordon Milson , James Morton , Brian Dawson , Greg Atkinson , and Daniel J. Green . 2011. “Influence of Cold Water Immersion on Limb and Cutaneous Blood Flow at Rest.” The American Journal of Sports Medicine 39(6): 1316–1323. 10.1177/0363546510395497. 21335348

[ejsc12074-bib-0025] Grgic, Jozo . 2023. “Effects of Post‐Exercise Cold‐Water Immersion on Resistance Training‐Induced Gains in Muscular Strength: A Meta‐Analysis.” European Journal of Sport Science 23(3): 372–380. 10.1080/15367290.2022.2033851. 35068365

[ejsc12074-bib-0026] Haun, Cody T. , Christopher G. Vann , Brandon M. Roberts , Andrew D. Vigotsky , Brad J. Schoenfeld , and Michael D. Roberts . 2019. “A Critical Evaluation of the Biological Construct Skeletal Muscle Hypertrophy: Size Matters but so does the Measurement.” Frontiers in Physiology 10: 247. 10.3389/fphys.2019.00247. 30930796 PMC6423469

[ejsc12074-bib-0067] Hohenauer, Erich , Jan Taeymans , Jean‐Pierre Baeyens , Peter Clarys , and Ron Clijsen . 2015. “The Effect of Post‐Exercise Cryotherapy on Recovery Characteristics: A Systematic Review and Meta‐Analysis.” PloS one 10(9): e0139028. 26413718 10.1371/journal.pone.0139028 PMC4586380

[ejsc12074-bib-0027] Horgan, Barry G. , Shona L. Halson , Eric J. Drinkwater , Nicholas P. West , Nicolin Tee , Rebekah D. Alcock , Dale W. Chapman , and G. Gregory Haff . 2023. “No Effect of Repeated Post‐Resistance Exercise Cold or Hot Water Immersion on In‐Season Body Composition and Performance Responses in Academy Rugby Players: A Randomised Controlled Cross‐Over Design.” European Journal of Applied Physiology 123(2): 351–359. 10.1007/s00421-022-05075-2. 36284024 PMC9895015

[ejsc12074-bib-0028] Horwath, Oscar , Helena Envall , Julia Röja , Eric B. Emanuelsson , Gema Sanz , Björn Ekblom , William Apró , and Marcus Moberg . 2021. “Variability in Vastus Lateralis Fiber Type Distribution, Fiber Size, and Myonuclear Content Along and Between the Legs.” Journal of Applied Physiology 131(1): 158–173. 10.1152/japplphysiol.00053.2021. 34013752

[ejsc12074-bib-0029] Ihsan, Mohammed , Chris R. Abbiss , and Robert Allan . 2021. “Adaptations to Post‐Exercise Cold Water Immersion: Friend, Foe, or Futile?” Frontiers in Sports and Active Living 3: 714148. 10.3389/fspor.2021.714148. 34337408 PMC8322530

[ejsc12074-bib-0030] Koh, T. J. , and F. X. Pizza . 2009. “Do Inflammatory Cells Influence Skeletal Muscle Hypertrophy?” Frontiers in Bioscience 1: 60–71. 10.2741/E7 19482625

[ejsc12074-bib-0031] Kraemer, William J. , and Nicholas A. Ratamess . 2005. “Hormonal Responses and Adaptations to Resistance Exercise and Training.” Sports Medicine 35(4): 339–361. 10.2165/00007256-200535040-00004. 15831061

[ejsc12074-bib-0032] Kruschke, John K. , and Torrin M. Liddell . 2018. “The Bayesian New Statistics: Hypothesis Testing, Estimation, Meta‐Analysis, and Power Analysis from a Bayesian Perspective.” Psychonomic Bulletin & Review 25(1): 178–206. 10.3758/s13423-016-1221-4. 28176294

[ejsc12074-bib-0033] Levine, James A. , Lana Abboud , Mitchel Barry , Judd E. Reed , Patrick F. Sheedy , and Michael D. Jensen . 2000. “Measuring Leg Muscle and Fat Mass in Humans: Comparison of CT and Dual‐Energy X‐Ray Absorptiometry.” Journal of Applied Physiology 88(2): 452–456. 10.1152/jappl.2000.88.2.452. 10658010

[ejsc12074-bib-0034] Madeyski, L. , and B. Kitchenham . 2018. “In: Effect Sizes and Their Variance for AB/BA Crossover Design Studies.” In Proceedings of the 40th International Conference on Software Engineering, 420. Gothenburg, Sweden. New York, NY: Association for Computing Machinery.

[ejsc12074-bib-0035] Malta, Elvis S. , Yago M. Dutra , James R. Broatch , David J. Bishop , and Alessandro M. Zagatto . 2021. “The Effects of Regular Cold‐Water Immersion Use on Training‐Induced Changes in Strength and Endurance Performance: A Systematic Review with Meta‐Analysis.” Sports Medicine 51(1): 161–174. 10.1007/s40279-020-01362-0. 33146851

[ejsc12074-bib-0036] Mawhinney, Chris , Helen Jones , David A. Low , Daniel J. Green , Glyn Howatson , and Warren Gregson . 2017. “Influence of Cold‐Water Immersion on Limb Blood Flow after Resistance Exercise.” European Journal of Sport Science 17(5): 519–529. 10.1080/15367290.2017.1279222. 28100130

[ejsc12074-bib-0037] McPhee, Jamie S. , and Adam P. Lightfoot . 2017. “Post‐Exercise Recovery Regimes: Blowing Hot and Cold.” The Journal of Physiology 595(3): 627–628. 10.1113/jp273503. 28145006 PMC5285623

[ejsc12074-bib-38] Moore, Emma , Joel T. Fuller , Jonathan D. Buckley , Siena Saunders , Shona L. Halson , James R. Broatch , and Clint R. Bellenger . 2022. “Impact of Cold‐Water Immersion Compared with Passive Recovery Following a Single Bout of Strenuous Exercise on Athletic Performance in Physically Active Participants: A Systematic Review with Meta‐analysis and Meta‐regression.” Sports medicine (Auckland, N.Z.) 52(7): 1667–1688. 35157264 10.1007/s40279-022-01644-9 PMC9213381

[ejsc12074-bib-0038] Morris, Scott B. 2008. “Estimating Effect Sizes from Pretest‐Posttest‐Control Group Designs.” Organizational Research Methods 11(2): 364–386. 10.1177/1094428106291059.

[ejsc12074-bib-0039] Morris, Scott B. , and Richard P. DeShon . 2002. “Combining Effect Size Estimates in Meta‐Analysis with Repeated Measures and Independent‐Groups Designs.” Psychological Methods 7(1): 105–125. 10.1037/1082-989x.7.1.105. 11928886

[ejsc12074-bib-0040] Morton, Robert W. , Kevin T. Murphy , Sean R. McKellar , Brad J. Schoenfeld , Menno Henselmans , Eric Helms , Alan A. Aragon , et al. 2017. “A Systematic Review, Meta‐Analysis and Meta‐Regression of the Effect of Protein Supplementation on Resistance Training‐Induced Gains in Muscle Mass and Strength in Healthy Adults.” British Journal of Sports Medicine 52(6): 376–384. 10.1136/bjsports-2017-097608. 31227491

[ejsc12074-bib-0041] Ogasawara, R. , and T. Suginohara . 2018. “Rapamycin‐insensitive Mechanistic Target of Rapamycin Regulates Basal and Resistance Exercise‐Induced Muscle Protein Synthesis.” The FASEB Journal: fj201701422R. 10.1096/fj.201701422R 29757673

[ejsc12074-bib-0042] Ohnishi, N. , M. Yamane , N. Uchiyama , S. Shirasawa , M. Kosaka , H. Shiono , and T. Okada . 2004. “Adaptive Changes in Muscular Performance and Circulation by Resistance Training with Regular Cold Application.” Journal of Thermal Biology 29(7–8): 839–843. 10.1016/j.jtherbio.2004.08.069.

[ejsc12074-bib-0043] Page, Matthew J. , Joanne E. McKenzie , Patrick M. Bossuyt , Isabelle Boutron , Tammy C. Hoffmann , Cynthia D. Mulrow , Larissa Shamseer , et al. 2021. “The PRISMA 2020 Statement: An Updated Guideline for Reporting Systematic Reviews.” BMJ 372: n71. 10.1136/bmj.n71. 33782057 PMC8005924

[ejsc12074-bib-0044] Peake, Jonathan M. , Llion A. Roberts , Vandre C. Figueiredo , Ingrid Egner , Simone Krog , Sigve N. Aas , Katsuhiko Suzuki , et al. 2017. “The Effects of Cold Water Immersion and Active Recovery on Inflammation and Cell Stress Responses in Human Skeletal Muscle after Resistance Exercise.” The Journal of Physiology 595(3): 695–711. 10.1113/jp272881. 27704555 PMC5285720

[ejsc12074-bib-0045] Petersen, Aaron C. , and Jackson J. Fyfe . 2021. “Post‐Exercise Cold Water Immersion Effects on Physiological Adaptations to Resistance Training and the Underlying Mechanisms in Skeletal Muscle: A Narrative Review.” Frontiers in Sports and Active Living 3: 660291. 10.3389/fspor.2021.660291. 33898988 PMC8060572

[ejsc12074-bib-0046] Petrofsky, J. S. , and M. Laymon . 2009. “Heat Transfer to Deep Tissue: The Effect of Body Fat and Heating Modality.” Journal of Medical Engineering & Technology 33(5): 337–348. 10.1080/03091900802069547. 19440919

[ejsc12074-bib-0047] Pointon, Monique , and Rob Duffield . 2012. “Cold Water Immersion Recovery After Simulated Collision Sport Exercise.” Medicine & Science in Sports & Exercise 44(2): 206–216. 10.1249/mss.0b013e31822b0977. 21716151

[ejsc12074-bib-0048] Poppendieck, Wigand , Melissa Wegmann , Anne Hecksteden , Alexander Darup , Jan Schimpchen , Sabrina Skorski , Alexander Ferrauti , Michael Kellmann , Mark Pfeiffer , and Tim Meyer . 2021. “Does Cold‐Water Immersion After Strength Training Attenuate Training Adaptation?” International Journal of Sports Physiology and Performance 16(2): 304–310. 10.1123/ijspp.2019-0965. 33217726

[ejsc12074-bib-0049] Resnick, B. , S. I. Zimmerman , D. Orwig , A. L. Furstenberg , and J. Magaziner . 2000. “Outcome Expectations for Exercise Scale: Utility and Psychometrics.” Journals of Gerontology Series B: Psychological Sciences and Social Sciences 55(6): S352–S356. 10.1093/geronb/55.6.s352. 11078112

[ejsc12074-bib-0050] Roberts, Llion A. , Kazunori Nosaka , Jeff S. Coombes , and Jonathan M. Peake . 2014. “Cold Water Immersion Enhances Recovery of Submaximal Muscle Function After Resistance Exercise.” American Journal of Physiology ‐ Regulatory, Integrative and Comparative Physiology 307(8): R998–R1008. 10.1152/ajpregu.00180.2014. 25121612

[ejsc12074-bib-0051] Roberts, Llion A. , Truls Raastad , James F. Markworth , Vandre C. Figueiredo , Ingrid M. Egner , Anthony Shield , David Cameron‐Smith , Jeff S. Coombes , and Jonathan M. Peake . 2015. “Post‐Exercise Cold Water Immersion Attenuates Acute Anabolic Signalling and Long‐Term Adaptations in Muscle to Strength Training.” The Journal of Physiology 593(18): 4285–4301. 10.1113/jp270570. 26174323 PMC4594298

[ejsc12074-bib-0052] Roux, Philippe P. , and John Blenis . 2004. “ERK and P38 MAPK‐Activated Protein Kinases: A Family of Protein Kinases with Diverse Biological Functions.” Microbiology and Molecular Biology Reviews 68(2): 320–344. 10.1128/mmbr.68.2.320-344.2004. 15187187 PMC419926

[ejsc12074-bib-0053] Schoenfeld, Brad Jon , and Alan Albert Aragon . 2018. “Is There a Postworkout Anabolic Window of Opportunity for Nutrient Consumption? Clearing Up Controversies.” Journal of Orthopaedic & Sports Physical Therapy 48(12): 911–914. 10.2519/jospt.2018.0615. 30702982

[ejsc12074-bib-0054] Swinton, Paul , and Andrew Murphy . 2022. “Comparative Effect Size Distributions in Strength and Conditioning and Implications for Future Research: A Meta‐Analysis.” SportRxiv. 10.51224/SRXIV.202.

[ejsc12074-bib-0055] Swinton, Paul Alan , Katherine Burgess , Andy Hall , Leon Greig , John Psyllas , Rodrigo Aspe , Patrick Maughan , and Andrew Murphy . 2022. “Interpreting Magnitude of Change in Strength and Conditioning: Effect Size Selection, Threshold Values and Bayesian Updating.” Journal of Sports Sciences 40(18): 2047–2054. 10.1080/02640414.2022.2128548. 36184114

[ejsc12074-bib-0056] Takarada, Yudai , Yutaka Nakamura , Seiji Aruga , Tetuya Onda , Seiji Miyazaki , and Naokata Ishii . 2000. “Rapid Increase in Plasma Growth Hormone After Low‐Intensity Resistance Exercise with Vascular Occlusion.” Journal of Applied Physiology 88(1): 61–65: Available from:. 10.1152/jappl.2000.88.1.61 10642363

[ejsc12074-bib-0057] Thorpe, Robin T. 2021. “Post‐Exercise Recovery: Cooling and Heating, a Periodized Approach.” Frontiers in Sports and Active Living 3: 707503. 10.3389/fspor.2021.707503. 34541521 PMC8440788

[ejsc12074-bib-0058] Timmerman, Kyle L. , Jessica L. Lee , Hans C. Dreyer , Shaheen Dhanani , Erin L. Glynn , Christopher S. Fry , Micah J. Drummond , Melinda Sheffield‐Moore , Blake B. Rasmussen , and Elena Volpi . 2010. “Insulin Stimulates Human Skeletal Muscle Protein Synthesis Via an Indirect Mechanism Involving Endothelial‐Dependent Vasodilation and Mammalian Target of Rapamycin Complex 1 Signaling.” The Journal of Cinical Endocrinology and Metabolism 95(8): 3848–3857. 10.1210/jc.2009-2696. PMC2913031 20484484

[ejsc12074-bib-0059] Tseng, C.‐Yu , Jo‐Ping Lee , Y.‐Shen Tsai , S.‐Da Lee , C.‐Lan Kao , Te‐Chih Liu , C.‐Hsiu Lai , M. Brennan Harris , and C.‐Hua Kuo . 2013. “Topical Cooling (Icing) Delays Recovery from Eccentric Exercise‐Induced Muscle Damage.” The Journal of Strength & Conditioning Research 27(5): 1354–1361. 10.1519/jsc.0b013e318267a22c. 22820210

[ejsc12074-bib-0060] Uchiyama, Shuichi , Hideo Tsukamoto , Shinichi Yoshimura , and Tetsuro Tamaki . 2006. “Relationship between Oxidative Stress in Muscle Tissue and Weight‐Lifting‐Induced Muscle Damage.” Pflügers Archiv 452(1): 109–116: Available from:. 10.1007/s00424-005-0012-y 16402246

[ejsc12074-bib-0061] Versey, Nathan G. , Shona L. Halson , and Brian T. Dawson . 2013. “Water Immersion Recovery for Athletes: Effect on Exercise Performance and Practical Recommendations.” Sports Medicine 43(11): 1101–1130. 10.1007/s40279-013-0063-8. 23743793

[ejsc12074-bib-0062] Wang, Yutan , Sijun Li , Yuanyuan Zhang , Yanru Chen , Fanghong Yan , Lin Han , and Yuxia Ma . 2021. “Heat and Cold Therapy Reduce Pain in Patients with Delayed Onset Muscle Soreness: A Systematic Review and Meta‐Analysis of 32 Randomized Controlled Trials.” Physical Therapy in Sport 48: 177–187. 10.1016/j.ptsp.2021.01.004. 33493991

[ejsc12074-bib-0063] Wilson, Laura J. , Lygeri Dimitriou , Frank A. Hills , Marcela B. Gondek , Aléchia van Wyk , Vlad Turek , Taylor Rivkin , et al. 2021. “Cold Water Immersion Offers No Functional or Perceptual Benefit Compared to a Sham Intervention During a Resistance Training Program.” The Journal of Strength & Conditioning Research 35(10): 2720–2727. 10.1519/jsc.0000000000004097. 34324460

[ejsc12074-bib-0064] Xiao, Feiyan , Anastasiia V. Kabachkova , Lu Jiao , Huan Zhao , and Leonid V. Kapilevich . 2023. “Effects of Cold Water Immersion After Exercise on Fatigue Recovery and Exercise Performance‐‐Meta Analysis.” Frontiers in Physiology 14: 1006512. 10.3389/fphys.2023.1006512. 36744038 PMC9896520

[ejsc12074-bib-0065] Yamane, M. , N. Ohnishi , and T. Matsumoto . 2015. “Does Regular Post‐exercise Cold Application Attenuate Trained Muscle Adaptation?” Int J Sports Med 36(8): 647–653. 10.1055/s-0034-1398652. 25760154

[ejsc12074-bib-0066] Yamane, Motoi , Hiroyasu Teruya , Masataka Nakano , Ryuji Ogai , Norikazu Ohnishi , and Mitsuo Kosaka . 2006. “Post‐Exercise Leg and Forearm Flexor Muscle Cooling in Humans Attenuates Endurance and Resistance Training Effects on Muscle Performance and on Circulatory Adaptation.” European Journal of Applied Physiology 96(5): 572–580. 10.1007/s00421-005-0095-3. 16372177

