# Supplementary material for: Throwing cold water on muscle growth: A systematic review with meta‐analysis of the effects of postexercise cold water immersion on resistance training‐induced hypertrophy
Source: Eur J Sport Sci. 2024 Feb 5;24(2):177–89. doi: 10.1002/ejsc.12074 (PMC11235606; doi:10.1002/ejsc.12074)
Supplement: Supplementary file 1 — Supplementary Material S1 [file EJSC-24-177-s002.pdf]

Supplementary Tables: Model details for Bayesian meta-analyses

Model details include the prior and posterior distribution of the meta-analysis parameters (pooled mean, additional uncertainty in level 1 within study variance due to unknown pre to post correlation values, level 2 between study variance and level 3 nested outcome variance) and probabilities that the pooled mean exceeded zero and small, medium, and large thresholds based on previous research conducted in strength and conditioning.

Table S1: Model details for preliminary analyses conducted on non-controlled effect sizes. Models include default weakly-informative prior distributions and informative prior distribution for additional level 1 within study variance.

	Mean [95% CrI]	Level 1: Within study calculated [75% CrI]	Level 1: Within study additional sigma [75% CrI]	Level 2: Between study [75% CrI]	Level 3: Study/Outcome [75% CrI]	Probability difference (>0)	Probability Small (>0.1)	Probability Medium (>0.35)	Probability Large (>0.7)
**Resistance training only**									
**Posterior**	0.36 [0.10 to 0.61]	0.33 [0.29 to 0.43]	0.08 [0.02 to 0.18]	0.15 [0.04 to 0.34]	0.11 [0.03 to 0.24]	0.995	0.977	0.541	0.007
**Prior**	Student_t(3,0.4,2.5)		Half Student_t(3,0,0.2)	Half Student_t(3,0,2.5)	Half Student_t(3,0,2.5)				
**Resistance training + cold water immersion**									
**Posterior**	0.14 [-0.08 to 0.36]	0.33 [0.27 to 0.39]	0.07 [0.02 to 0.16]	0.10 [0.02 to 0.25]	0.09 [0.02 to 0.19]	0.906	0.668	0.029	<0.001
**Prior**	Student_t(3,0.4,2.5)		Half Student_t(3,0,0.2)	Half Student_t(3,0,2.5)	Half Student_t(3,0,2.5)				

Table S2: Model details for primary analyses conducted on comparative effect sizes. Models include default weakly-informative prior distributions and informative prior distribution for additional level 1 within study variance and informative prior distribution for the pooled mean based on previous research in strength and conditioning. A sensitivity analysis including a default weakly-informative prior distribution for the pooled mean was also included.

	**Mean [95% CrI]**	**Level 1: Within study calculated [75% CrI]**	**Level 1: Within study additional sigma [75% CrI]**	**Level 2: Between study [75% CrI]**	**Level 3: Study/Outcome [75% CrI]**	**Probability difference (<0)**	**Probability Small (<-0.1)**	**Probability Medium (<-0.3)**	**Probability Large (<-0.5)**
**Resistance training only compared with resistance training + cold water immersion (Informative prior for pooled mean)**									
**Posterior**	-0.22 [-0.47 to 0.04]	0.40 [0.25 to 0.45]	0.09 [0.02 to 0.23]	0.12 [0.03 to 0.29]	0.13 [0.03 to 0.31]	0.957	0.834	0.254	0.018
**Prior**	Normal(0,0.43^2)		Half Student_t(3,0,0.2)	Half Student_t(3,0,2.5)	Half Student_t(3,0,2.5)				
**Resistance training only compared with resistance training + cold water immersion (Weakly-informative prior for pooled mean)**									
**Posterior**	-0.25 [-0.52 to 0.01]	0.40 [0.25 to 0.45]	0.10 [0.02 to 0.22]	0.12 [0.02 to 0.27]	0.14 [0.03 to 0.31]	0.969	0.888	0.336	0.031
**Prior**	Student_t(3,-0.1,2.5)		Half Student_t(3,0,0.2)	Half Student_t(3,0,2.5)	Half Student_t(3,0,2.5)				

Table S3: Model details for meta-regression assessing the moderation effect of intervention duration and training status on comparative effect sizes. Model includes default weakly-informative prior distributions and informative prior distribution for additional level 1 within study variance.

	Difference across levels [95% CrI]	Level 1: Within study calculated [75% CrI]	Level 1: Within study additional sigma [75% CrI]	Level 2: Between study [75% CrI]	Level 3: Study/Outcome [75% CrI]	Probability difference (<0)
**Intervention duration (Shorter [<8 weeks] to Longer [≥8 weeks])**						
**Posterior**	-0.04 [-0.61 to 0.55]	0.40 [0.25 to 0.45]	0.10 [0.02 to 0.23]	0.14 [0.03 to 0.29]	0.16 [0.04 to 0.34]	0.570
**Prior**	Flat prior		Half Student_t(3,0,0.2)	Half Student_t(3,0,2.5)	Half Student_t(3,0,2.5)	
**Training status (Trained to Untrained)**						
**Posterior**	-0.10 [-0.65 to 0.43]	0.40 [0.25 to 0.45]	0.10 [0.02 to 0.23]	0.14 [0.03 to 0.29]	0.16 [0.04 to 0.34]	0.653
**Prior**	Flat prior		Half Student_t(3,0,0.2)	Half Student_t(3,0,2.5)	Half Student_t(3,0,2.5)	
