# Supplementary material for: Throwing cold water on muscle growth: A systematic review with meta‐analysis of the effects of postexercise cold water immersion on resistance training‐induced hypertrophy
Source: Eur J Sport Sci. 2024 Feb 5;24(2):177–89. doi: 10.1002/ejsc.12074 (PMC11235606; doi:10.1002/ejsc.12074)

PRISMA 2020 flow diagram for new systematic reviews which included searches of databases and registers only

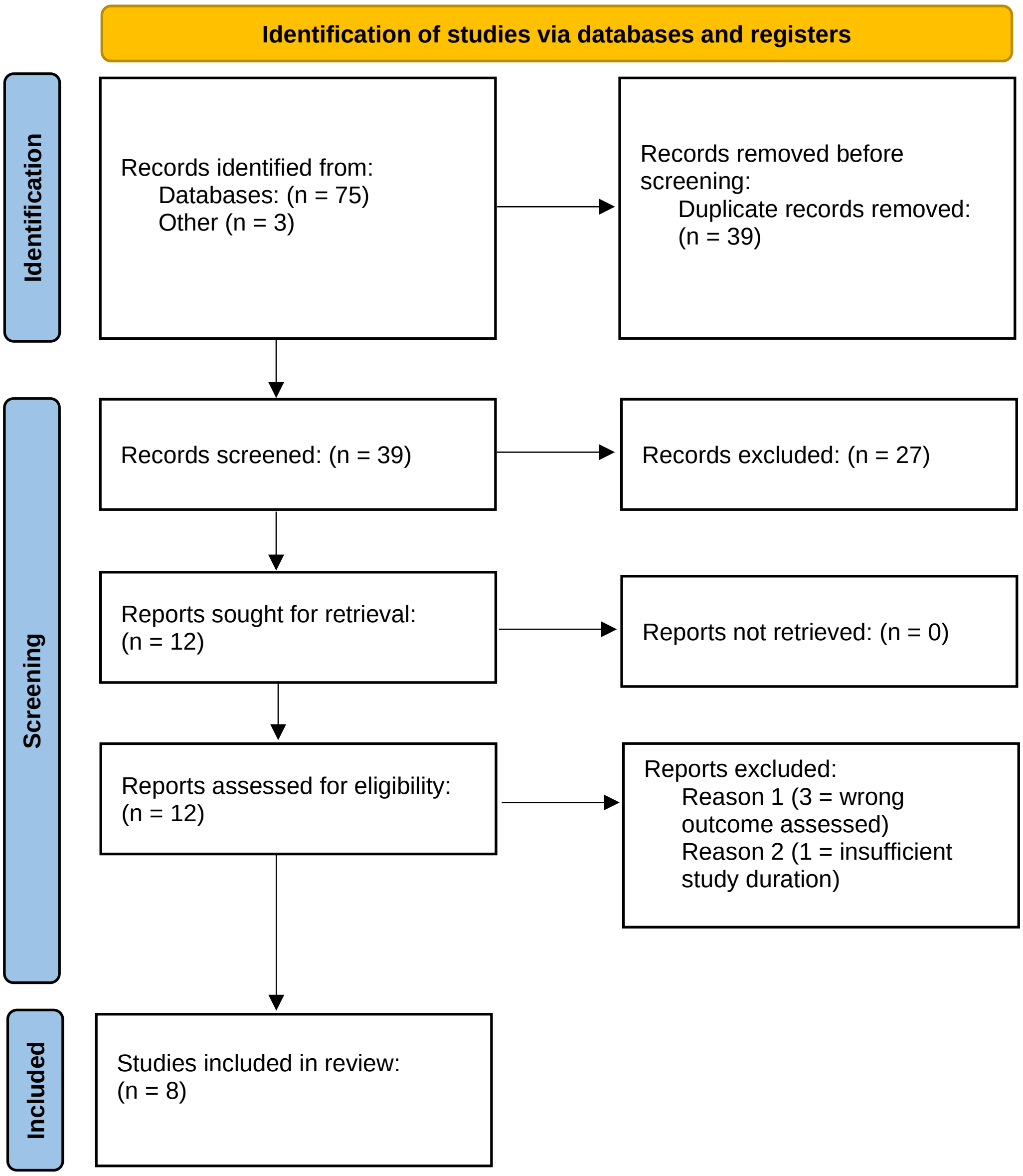

Supplement: Supplementary file 2 — Figure S1 [file EJSC-24-177-s001.pdf]
